# Tool to assess the groundstroke technique of preadolescent tennis players

**DOI:** 10.3389/fspor.2024.1341138

**Published:** 2024-04-05

**Authors:** Hakan Diler, Asuman Şahan, Kemal Alparslan Erman

**Affiliations:** ^1^ Institute of Medical Science, Akdeniz University, Antalya, Turkey; ^2^ Faculty of Sport Sciences, Akdeniz University, Antalya, Turkey

**Keywords:** tennis, technique, groundstroke, performance assessment, test reliability

## Abstract

**Objective:**

In this study, we develop a tool that can be used by tennis coaches to evaluate the groundstroke (forehand and backhand) technique of preadolescent tennis players.

**Methods:**

The participants of the study were 60 children (30 males and 30 females) aged 10–12 years, with at least two years of training in tennis. The Groundstroke Correction Checklist (GCC) was translated into Turkish by using a blind procedure. The Turkish translation was then evaluated by 15 coaches of the Turkish Tennis Federation who had at least a level-3 coaching license and more than five years of coaching experience. The technical components related to technique in the checklist were labeled as unimportant, important, and very important. Following this, the GCC was converted into a Groundstroke Technique Assessment Test Tool (GTATT) by a selection committee consisting of three experts, and its reliability and validity were assessed by using it in the field. Spearman's correlation was used to analyze the correlation (test-*r* test) between the technical evaluation scores assigned to the players by the tennis coaches based on the GTATT in the first and second weeks. Intra- and inter-rater reliability was used to analyze the overall scores of technical evaluations in the first and second weeks to assess the reliability of the scale used. We determined each player's number of years of playing experience (TPY), technical evaluation (TE), international tennis-level test score (ITN), I-cord classification order (ICCO), and the number of games won (GW) in a tournament organized among themselves and evaluated the correlations among these parameters by using Spearman's correlation analysis.

**Conclusion:**

A statistically high and significant correlation was observed between the technical evaluations of the players' forehand and backhand groundstrokes by the tennis coaches by using the GTATT in the first and second weeks (*r* > .90, *p* < .01). The analysis of the intra- and inter-rater reliability of the GTATT yielded excellent agreement between the technical observations of the three coaches of the players' forehand and backhand strokes in the first and second weeks.

## 1 Introduction

It is believed that technical evaluation based on objective criteria and tools of measurement can enable players to improve the quality of their strokes in training, and the improved technique has an indirect positive effect on their performance in competitions. Such an evaluation also makes it possible to measure and assess the technical development of players.

Tennis is a globally popular sport that is played by millions of people at different levels of skill (1). As in many other sports, the interactions among the technical, anthropometric, physiological, tactical, and psychological characteristics tennis players have an important influence on their performance and success (2).

Tennis has a competitive identity. The technical and tactical capacity of the player are among the most important factors influencing the outcome of matches. The totality of technical and tactical actions, together with the competitive ability of the players, influence the result (3). Forehand and backhand strokes are the most common shots played in tennis competitions (4), and are the two most important strokes after the service stroke (5).

The relevant literature has shown that it is possible to attain the desired movement patterns and ensure skill development in sport by ignoring the influence of developments in motor learning based on changes in the coordination of movement on the resulting performance and/or subjective evaluations of the patterns of movement (6). However, it is important for coaches to easily conduct accurate technical evaluations to create the desired movement patterns.

A combination of technical and tactical skills is more likely than other factors to help distinguish between players with different levels of performance (7). In tennis, a player can enhance their tactical diversity with a technique that is simple, effective, and economical by using the appropriate biomechanical principles. In terms of biomechanics, the inefficient movements and positioning of various joints can adversely affect the speed, direction, and rotation of the ball, and may also increase the risk of injury to the player (8). Technique in tennis is generally characterized by the quality of the stroke. The critical elements that determine the quality of the stroke are the ball speed, and the accuracy and rate of success of the stroke (9). The constant interplay between technical and tactical skills is critical to earning each point in a match. We can consider technique as a functional tool for achieving a tactical goal. For example, a player who chooses to move their opponent out of the court as a tactical goal needs to hit the ball with the appropriate speed, precision, and angle. Moreover, a player playing against an opponent who is aggressively hitting deep balls toward the back line can gain the time they need to recover by throwing high and deep balls. In such cases, the techniques that the player uses in their tactical decisions come to the fore (10).

As in other sports, technique is the basis for player development in tennis, and all the coach's efforts will be futile if the player does not internalize the correct technique. Incorrect neural and movement-related patterns are stored in the brain if the technical training procedures are incorrectly applied, and severely limit the player's subsequent development of their desired optimum speed. For instance, incorrect patterns can affect the player's batting speed and accuracy (11).

Technical training should be an important part of the training cycle for junior players (6–12 years old), which in turn should be built on sound foundations when they are 5–10 years old. As players get older, the technical aspect of their training diminishes, such that more minor adjustments and interventions are required. When they are 12–16 years old, their technique should be improved through deliberate practice. In other words, their technique should be reinforced through conscious practice (1).

A tool with proven reliability and validity for the technical evaluation of the forehand and backhand, which are the most frequently used groundstrokes in a tennis match, has not been proposed in the literature to the best of our knowledge. It is thought that technical evaluation based on objective criteria and tools of measurement can enable players to enhance the quality of their strokes in training, and this has an indirect positive effect on their performance in competition. It also offers the possibility of measuring and evaluating the technical development of players. Furthermore, using common criteria to assess the player's movements while hitting the ball can transform such a technical evaluation from being grounded in subjective criteria to being based on more objective features. In light of this, we develop a test tool in this study that can be used by tennis coaches to evaluate the groundstroke (forehand and backhand) technique of players as this constitutes the basic technical skill in the game. We think that our tool can enable tennis coaches to easily, quickly, and accurately evaluate the groundstroke technique of player on the court without requiring additional equipment or materials.

## 2 Materials and methods

### 2.1 Participants

The criteria for the inclusion of the subjects in our experiments were a minimum of two years of experience playing tennis, active participation in tennis training, absence of health problems, and no medication use. Participation in the study was voluntary.

Sixty tennis players (30 males and 30 females) (age 11.47 ± 0.89 years; body height 143.97 ± 8.68 cm; weight 40.85 ± 6.49 kg) with at least two years of tennis training and an average experience of 3.50 ± 0.82 years voluntarily participated in the study. All participants were training four to six times a week at the time of the experiment. The test–retest reliability of the Groundstroke Technique Assessment Test Tool was assessed on these 60 tennis players. We also recruited 15 coaches who met the following criteria: at least a level-3 coaching certificate from the Turkish Tennis Federation (TTF), professional experience of 18.66 ± 3.47 years, with an average age of 44.73 ± 6.15 years, record of service as a national team coach.

### 2.2 Procedures

All parents/guardians were informed of the experimental procedures as well as the associated risks prior to providing their written consent. The study was approved by the Akdeniz University Clinical Research Ethics Committee (No. 70904504/276). All procedures were performed in accordance with the Declaration of Helsinki (1964).

The Groundstroke Technique Assessment Test Tool (GTATT) was developed in the following three phase:

Phase 1: The International Tennis Federation's (ITF) groundstroke correction checklist (GCC) (10) was translated into Turkish by two tennis coaches who had an advanced knowledge of English. The final Turkish version of the tool was subsequently translated back into English by a native English speaker. The translation involved a blind procedure. The translated checklist (GC) was then compared with the original version, and the two were determined to have very similar meanings.

Phase 2: The translated version of the GCC was sent to the coaches via email. They were asked to mark the parameters on the observation evaluation form that they considered the most important for the groundstrokes of 10–12-year-old players by using the following options: unimportant, important, and very important.

Phase 3: According to the evaluations provided by the 15 coaches, the items marked as very important and important among the parameters of the forehand and backhand strokes were examined by a selection committee comprising three experts.

The most commonly marked items by the coaches and those developed by the selection committee were identified according to the results of the review and were used to create the GTATT (Table 1).

**Table 1:** Grading scale for the groundstroke technique assessment test tool.

Grading scale for the groundstroke technique assessment test tool			
Player's given name and surname:			
Age:			
Trainer's given name and surname:			
Date:			
Forehand:	Backhand:	Rating	
Evaluation topic	Weak	Middle	Good
(1) Racket grip			
(2) Ready position			
(3) Split step			
(4) Shoulder rotation			
(5) Footwork			
(6) Racket reversal			
(7) Bending the racket head and wrist under the ball			
(8) Ball meeting point			
(9) Racket speed			
(10) Rhythm and path of the racket			
(11) Balance during the stroke			
(12) Footwork and recovery			

Before using the GTATT, which consisted of 12 stages, the coaches read the criteria for technical evaluation (Table 2) and then took their places on the field.

**Table 2:** Criteria used for technical evaluation.

	Forehand groundstroke	Backhand groundstroke
1	Eastern forehand Semi-western Western	Single hand (Eastern Backhand, Continental) Double hand (Right-hand continental; left-hand eastern forehand) (Right-hand eastern forehand; left-hand eastern forehand)
2	1-The center of gravity of the body is at the level of and inside the soles of the feet. 2-Head and shoulders are aligned so that they are straight. 3-The upper body bends slightly forward at the waist. The knees are energized by being slightly bent. 4-Feet spread out to shoulder level. Body weight is kept on the toes, staying in front. The center of gravity is lower and more anterior than normal.	
3	As the opponent hits the ball, a forward rise is attained by bending the knee joints and lifting the ankles off the ground.	
4	1-The unit spin can start as soon as the turn preparation jump (split step) is completed. 2-An outward lunge is made by using the foot closest to the ball. This move also initiates the backward racket movement through the rotation of the hips and shoulders. 3-The racket arm is used to push the body backward in a unit spin. 4-It is important not to move the head and shoulders forward or step backward with the front foot.	
5	1-The unit spin can start immediately after a split step with two feet. 2-Stepping out is performed by using the foot closest to the ball. The direction of this fast, lateral movement and step should be diagonal and forward with respect to the incoming ball. 3-Again, this movement initiates the backward movement of the racket through the rotation of the hips and shoulders. 4-The racket arm is used to push the back during the unit spin. Do not move forward with the head and shoulders or step back with the front foot. The player is as fast as their speed in the first step.	
6	Eastern Forehand 1-The racket handle is positioned under the head of the racket, and its face is positioned more freely upward. As the racket is moved backward in a circle, the racket head also moves backward. The body weight is transferred to the back foot. Semi-Western Forehand 1-In general, preparation starts with the elbow. 2-As the opponent hits the ball, rotate around the front foot and continue by raising the elbow (backward movement), rotating the shoulders in a coordinated manner. 3-The left hand can be used to pull the racket back and contribute to shoulder rotation.	Double-hand backhand 1-Shoulder rotations occur automatically when the racket is pulled backward with both hands. 2-The hips and upper body turn back in the phase before the front step is taken. The player transfers linear momentum with the step forward. 3-The racket is lifted behind the body. The rotation of the hips begins. One-hand backhand 1-The player rotates from the ready position, as the hand and racket are in one place as the ball approaches. 2-Either of two methods can be used when approaching the end of the racket retrieval phase. First, the racket is lifted to shoulder height, rotated, the hand is taken back, and the position is assumed. Second, the shoulder is rotated (in a half-U shape) and, immediately afterward, the hand is raised and the back swing curve is performed.
7	According to the height of the incoming ball, the racket and the wrist are lowered below its level before it is hit, and the movement is assisted by bending the knees.	
8	The racket is expected to meet the ball in front of and to the side of the body. The contact point with Western grips is higher and closer to the body than with Eastern and semi-Western grips.	The racket is expected to meet the ball in front of and to the side of the body. The contact point with Western grips is higher and closer to the body than with Eastern and semi-Western grips. During the stroke, the ball is struck in front of the front foot.
9	The racket speed is as high as possible at the point of contact, and is adjusted for the purpose of the stroke.	
10	Once the acceleration phase of the racket has begun, it is necessary to move it along the right path. The path of the racket immediately before and after hitting the ball is a straight line.	
11	Good balance during the stroke requires that the head be upright and still, and the head and upper body move. It is also helpful to keep the arm at a comfortable distance from the body during the forward swing to maintain control over the head of the racket when hitting the ball, and to hit the ball without leaving the line of flight.	
12	The body weight is shifted forward, and the arm is moved forward toward the target in a relaxed state. During the pursuit, there is a gradual deceleration of the body parts. At the same time, if the stroke has a sufficiently high linear acceleration, the back leg moves to prepare the movement for the next stroke by assuming a position with the foot in front.	

Previous studies have used a five-point scale to assess the technique of children in tennis (12). The five-point Likert scale is a widely used tool in the social sciences to capture attitudes, opinions, and perceptions. It contains a series of statements or questions that respondents are asked to rate on a five-point scale ranging from “totally agree” to “strongly disagree.

Warming up:

The players warmed up for 10 min in the traditional manner used during training, consisting of 5 min of low-paced running and 5 min of dynamic stretching. Then, two players warmed up by rallying, first from the service line and then from the base line, within the 5-min warm-up period implemented by the TTF for official matches.

Technical evaluation protocol:

The GTATT was applied on the tennis court for the 10–12-year-old players by three coaches, each with at least 10 years of professional experience as a level-3 trainer. The technical evaluation was conducted on a hard-floor indoor tennis court with the regular dimensions as determined by the ITF.

The balls were fed to the players from the opposite side of the court by using the SAM P1 ball-throwing machine. ITF-approved tennis balls, fresh from a pressurized box, were used for the evaluation. The duration between forehand and backhand strokes was set to 2 s, the speed to two units, and the amount of spin in the forward direction to four units for the ball thrower. The balls were thrown at a moderate velocity (40 km.h^−1^).

Previous studies have reported that the rally pace (mean duration of flight of the ball between opponents) in tennis matches varies depending on the playing surface. The historical rally pace is the highest in the Australian Open (1.22 s), significantly higher than that in the French Open (1.35 s). The rally pace in Wimbledon is 1.27 s for the men's tournament (13). We set the rally pace to 2 s in order to avoiding affecting the players' technique as they hit the ball.

The players started hitting the balls thrown by the ball machine from behind the base line on the court to a designated 90 cm^2^ area. Each player was then asked to hit the balls toward a marked 3 m area in front of the baseline. This instruction was given only to motivate the players to focus on a specific area, rather than resort to random hits. Each player played forehand and backhand strokes in the same manner toward the marked area until all three coaches had filled out the GTATT and declared that they had completed their observations. The evaluation then ended. The coaches observed the players from positions of their own choosing so that they could not see one another's evaluations. We ensured that there was no contact between the coaches during the evaluation. The speed and accuracy with which the balls were hit were not considered in the GTATT.

Implementation of international tennis level (ITN) test:

The international tennis number (ITN) manual on-court assessment test was introduced by the ITF to rate the skill of players. The test involves measurements of the accuracy and depth of the groundstroke, service accuracy, depth of the volley, and the mobility of the player. We used only the depth–power (groundstroke depth assessment) and accuracy–power (groundstroke precision assessment) sections of the ITN test in this study (14).

### 2.3 Test of groundstroke depth

**Figure 1:**
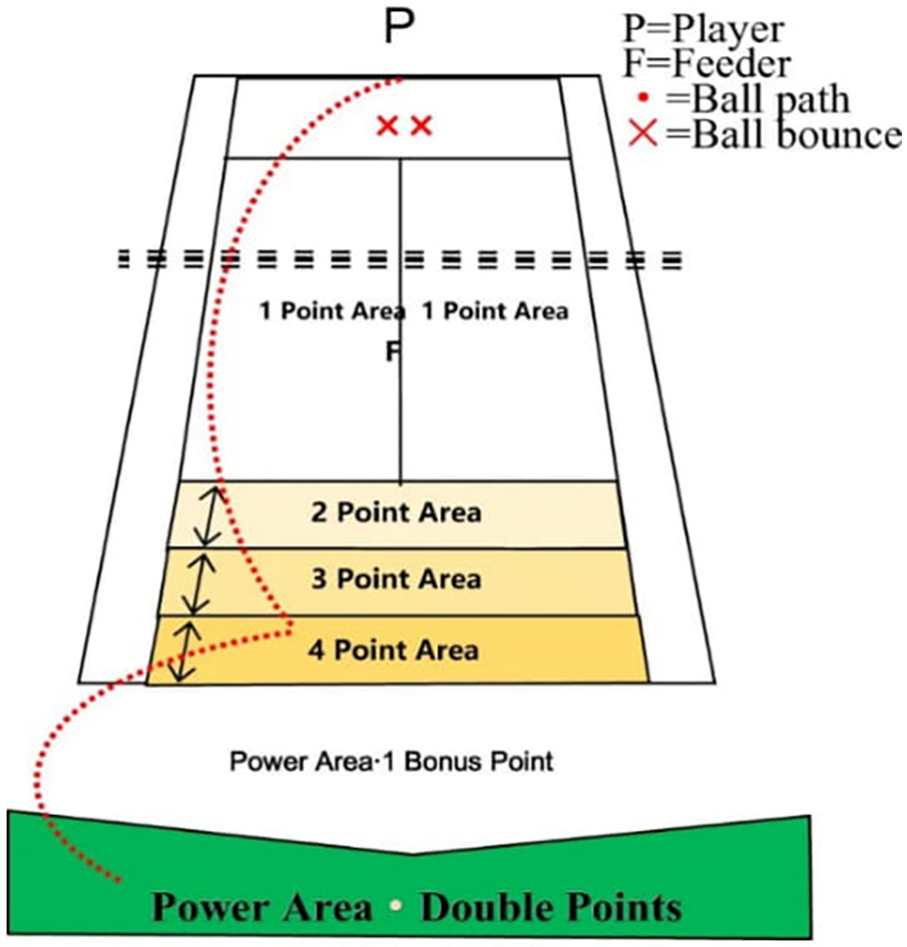
Test of groundstroke depth. (Available online at: https://tenis.i-kort.ttf.org.tr/home.seam)

The manner in which this test was performed is shown in Figure 1, where “*P*” represents the player and “*F*” the feeder. To evaluate the test, 10 top feeds were made toward the area marked with “*x x*” in the figure in front of the breakthrough balls of *F* (*P*). The player was required to hit five balls using the forehand stroke and the other five by using the backhand stroke. The player received zero points when a ball's first bounce lands anywhere outside the normal singles playing area.

(1) The player was awarded one to four points depending on the first region in which the ball fell.
(2) According to the region in which the ball fell, the player was awarded zero points if the ball fell in their own half of the tennis court.

Power Area = 1 Bonus Point—When a ball lands anywhere within the singles court area and the second bounce lands between the baseline and Bonus line, 1 Bonus point is awarded.

Power Area = Double Points—When a ball lands anywhere within the singles court area and the second bounce lands beyond the Bonus line, double points are awarded.

Each player could earn a maximum of 90 points in this part of the test (Figure 1).

### 2.4 Test of groundstroke accuracy

**Figure 2:**
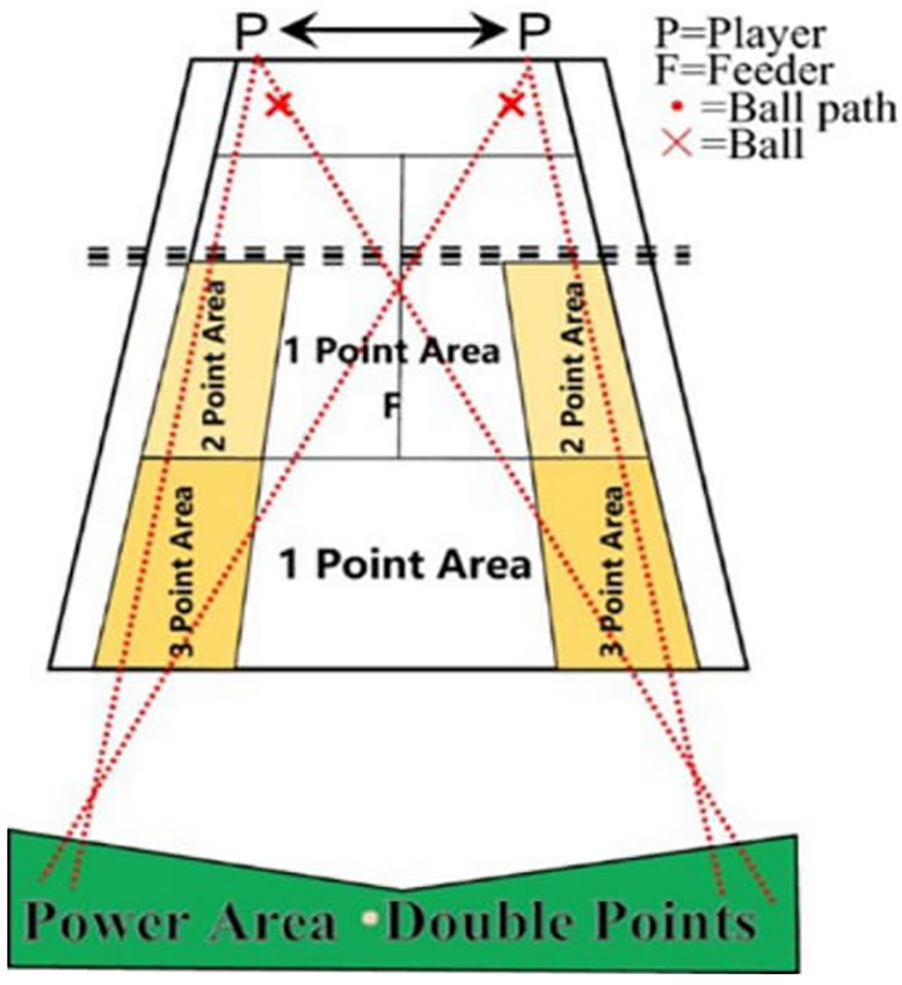
Test of groundstroke accuracy. (Available online at: https://tenis.i-kort.ttf.org.tr/home.seam)

The ball machine F alternately threw six balls, to either side of the player for them to play the forehand and backhand strokes, respectively, to the areas denoted by “*x*” in Figure 2. The player throws six more balls alternately, one in front of the hand and one in the back of the hand, towards the places indicated by the letters “*x x*” in front of him. The player throws these balls diagonally.

The player received zero points if the ball went out of bounds or was caught in the net and was otherwise awarded one to three points depending on where the ball first landed.

The player was awarded zero points if the ball landed in their half of the court.

Power Area = 1 Bonus Point—When a ball lands anywhere within the singles court area and the second bounce lands between the Baseline and Bonus line, 1 bonus point is awarded.

Power Area = Double Points—When a ball lands anywhere within the singles court area and the second bounce lands beyond the Bonus line, double points are awarded. Each player could earn a maximum of 84 points in this part of the experiment (Figure 2).

I-cord classification order (ICCO) score:

The players are ranked in this age category according to their performance in national and international tournaments in the preceding 52 weeks. We obtained the general classification from the TTF's official website (15).

Games won (GW):

To determine their competitive performance, we asked 30 players in the 10-, 11-, and 12-year age categories to play in groups of five in accordance with the instructions of the TTF for competitive tournaments for two short (four-game) sets. Male and female players competed separately. In case each player won a set each, a 10-point match was played as a tiebreaker. The groups of five players each were determined by draw for players in each age category. New balls were used for these matches. At the end of the competition, the total number of games won by each player in all their matches was calculated.

### 2.5 Statistical analyses

The number of players required to obtain a significant effect was calculated by using Gpower 3.1.9.4 (16). The appropriate sample size was determined to be 67 for a 5% margin of error and an 80% confidence interval, with the correlation coefficient *r* = 0.3. As mentioned above, our experiment involved 60 players.

To determine the homogeneity of the data obtained in this study, we used the Kolmogorov–Smirnov test, which is a test of the normal distribution (because the number of subjects was greater than 50). All data were analyzed by using the SPSS 25 package. Descriptive statistical values, such as the arithmetic mean and standard deviation (x¯ ± SD), were calculated for all variables. A margin of error of *α* = .05 was used in all statistical procedures. Data on the descriptive characteristics of the participants were presented as the arithmetic mean ± standard deviation (Mean ± SS).

To assess test-retest reliability, Spearman's correlation or intraclass correlation coefficient (ICC) was calculated (17, 18). Spearman's correlation coefficient was applied for the correlation analysis (test–retest) between the technical evaluation scores of the tennis coaches in the study in the first and second weeks. Differing from Spearman's correlation coefficient interpretation, intra- and inter-rater reliability was performed. Results were analyzed ICC 2-way mixed effects model to determine intra- and inter- rater reliability of the Groundstroke Technique Assessment Test Tool. ICC values of less than 0.4, between 0.4 and 0.59, between 0.60 and 0.74, and above 0.74 are indicator of poor, fair, good, and excellent agreement respectively.

The relations between the following parameters, obtained from the male and female tennis players, were determined by using Spearman's correlation analysis: the player's years of playing (TPY), technical evaluation (TE), international tennis-level test score (ITN), I-cord classification order (ICCO), and number of games won (GW). The values of r were evaluated as follows in terms of correlation: *r* < 0.50 = weak, *r* > 0.50 = moderate, *r* > 0.75 = good, and *r* > 0.90 = high (19).

## 3 Results

Table 3 shows test points of the players participating in the study.

**Table 3:** Test points of participants.

Variables	Min.	Max.	Mean (SD)
TPY (years)	2.00	6.00	3.50 (0.77)
TE (points)	23.33	36.00	31.09 (3.09)
GDA (points)	24.00	74.00	43.65 (10.62)
GPA (points)	26.00	69.00	51.18 (10.58)
ITN (points)	31.00	131.00	94.35 (17.70)
ICCO (points)	190.00	2,364.00	985.73 (528.36)
GW (points)	8.00	33.00	22.20 (7.97)

TPY, years of playing tennis; TE, technical evaluation; GDA, groundstroke depth assessment; GPA, groundstroke precision assessment; ITN, international tennis-level test score; ICCO, I-cord classification order; GW, games won.

Table 4 shows that according to Spearman's rho (*r*) correlation analysis, a statistically positive, high, and significant correlation was found between the first- and second-week technical observations of the three observer coaches for the forehand and backhand strokes.

**Table 4:** Spearman's correlation analysis of observer coaches’ (*n* = 60) technical observation of players’ ground strokes in the first and second weeks.

Variable rho (*p*)	FH1W1C	FH2W1C	BH1W1C	BH2W1C	FH1W2C	FH2W2C	BH1W2C	BH2W2C	FH1W3C	FH2W3C	BH1W3C
FH2W1C	.94** (.001)**	–									
BH1W1C	.94** (.001)**	.96** (.001)**	–								
BH2W1C	.92** (.001)**	.96** (.001)**	.97** (.001)**	–							
FH1W2C	.91** (.001)**	.95** (.001)**	.95** (.001)**	.95** (.001)**	–						
FH2W2C	.85** (.001)**	.91** (.001)**	.88** (.001)**	.88** (.001)**	.90** (.001)**	–					
BH1W2C	.90** (.001)**	.92** (.001)**	.94** (.001)**	.91** (.001)**	.92** (.001)**	.87** (.001)**	–				
BH2W2C	.88** (.001)**	.88** (.001)**	.91** (.001)**	.90** (.001)**	.91** (.001)**	.83** (.001)**	.95** (.001)**	–			
FH1W3C	.88** (.001)**	.90** (.001)**	.90** (.001)**	.90** (.001)**	.90** (.001)**	.82** (.001)**	.90** (.001)**	.90** (.001)**	–		
FH2W3C	.83** (.001)**	.90** (.001)**	.90** (.001)**	.90** (.001)**	.90** (.001)**	.84** (.001)**	.90** (.001)**	.90** (.001)**	.97** (.001)**	–	
BH1W3C	.88** (.00)**	.91** (.001)**	.91** (.001)**	.90** (.001)**	.92** (.001)**	.83** (.001)**	.92** (.001)**	.90** (.001)**	.94** (.001)**	.94** (.001)**	–
BH2W3C	.90** (.001)**	.92** (.001)**	.94** (.001)**	.92** (.00)**	.91** (.001)**	.83** (.001)**	.93** (.001)**	.91** (.001)**	.93** (.001)**	.94** (.001)**	.97** (.001)**

FH, forehand; BH, backhand; 1W, first week; 2W, second week; 3W, third week; 1C, first coach; 2C, second coach; 3C, third coach.

** Correlation is significant at the 0.01 level.

Table 5 shows that the analysis of the intra- and inter-rater reliability of the GTATT yielded excellent agreement between the technical observations of the three coaches of the players' forehand and backhand strokes in the first and second weeks.

**Table 5:** Intra- and inter-rater reliability of the technical observation tool according to scores.

Intra-rater reliability	ICC	95% CI
FH1W1C- FH2W1C	0.98	0.97–0.99
BH1W1C- BH2W1C	0.99	0.99–0.99
FH1W2C- FH2W2C	0.99	0.98–0.99
BH1W2C- BH2W2C	0.99	0.98–0.99
FH1W3C- FH2W3C	0.99	0.98–0.99
BH1W3C- BH2W3C	0.99	0.98–0.99
Inter-rater reliability		
FH 1W1-2-3C	0.97	0.95–0.98
FH 2W1-2-3C	0.97	0.96–0.98
BH 1W1-2-3C	0.98	0.97–0.99
BH 2W1-2-3C	0.98	0.97–0.99

FH, forehand; BH, backhand; 1W, first week; 2W, second week; 3W, third week; 1C, first coach; 2C, second coach; 3C, third coach; ICC, intra-class correlation coefficient (interpretation: <0.40 poor; 0.40–0.59 fair; 0.60–0.74 good; >0.74 excellent). CI, confidence interval.

According to Table 6, a positive and moderately significant correlation was observed between GDA and TE (**p < .001, *r* > .50). Further, there was a moderately significant positive correlation between GPA and TE (**p < .001, *r* > .50), and a positive and significant correlation between ITN and TE (**p < .001, *r* > .75). There was a negative and significant correlation between ICCO and TE (**p < .01, *r* > .75). A positive and moderately significant correlation was identified between GW and TE (**p < .001, *r* > .50).

**Table 6:** Results of the analysis of correlations between the variables of male athletes.

Variables	TPY	GDA	GPA	ITN	ICCO	GW
GDA (points)	.44	–				
	(.01)**	–				
GPA (points)	.29	.53	–			
	(.12)	(.001)**	–			
ITN (points)	.43	.87	.87	–		
	(.01)**	(.001)**	(.001)**	–		
ICCO (points)	-.26	-.70	-.72	-.80	–	
	(.16)	(.001)**	(.001)**	(.001)**	–	
GW (points)	.44	0.80	0.55	.77	-.57	–
	(.09)	(.001)**	(.03)*	(.001)**	(.02)*	–
TE (points)	.29	.69	.61	.75	-.86	.68
	(.11)	(.001)**	(.001)**	(.001)**	(.001)**	(.001)**

* *p* < .05.

** *p* < .01.

TPY: Years of playing. TE: Technical evaluation. GDA: Groundstroke depth assessment. GPA: Groundstroke precision assessment. ITN: International tennis-level test score. ICCO: I-cord classification order. GW: Games won.

Table 7 shows a positive and moderately significant correlation between GDA and TE (**p < .001, *r* > .50), and a weakly significant positive correlation between GPA and TE (*p < .05, *r* < .50). A negative and moderately significant correlation was observed between ITN and TE (**p < .001, *r* > .50). A negative and moderately significant relation was identified between ICCO and TE (**p < .01, *r* > .50). Moreover, a positive and moderately significant relation was found between GW and TE (**p < .01, *r* > .50).

**Table 7:** Results of the analysis of correlations between the variables of female athletes.

Variables	TPY	GDA	GPA	ITN	ICCO	GW
GDA (points)	.15	–				
	(.40)	–				
GPA (points)	.25	.53	–			
	(.17)	(.78)	–			
ITN (points)	.30	.71	.66	–		
	(.10)	(.001)**	(.001)**	–		
ICCO (points)	-.61	-.51	-.52	-.70	–	
	(.001)**	(.001)**	(.001)**	(.001)**	–	
GW (points)	.35	.47	.89	.30	-.59	–
	(.09)	(.001)**	(.03)*	(.27)	(.02)*	–
TE (points)	.45	.64	.36	.70	-.72	.70
	(.01)	(.001)**	(.04)*	(.001)**	(.001)**	(.001)**

* *p* < .05.

** *p* < .01.

## 4 Discussion

We have developed a test tool that can be used by tennis coaches to evaluate the groundstroke (forehand and backhand) technique of preadolescent tennis players. It is difficult to analyze the technique of players at forehand and backhand strokes, which are the most commonly used shots in tennis, without video recordings, especially in a competitive environment. The players' stroke technique during a match is influenced by the opponent's choice of stroke, the speed of arrival of the ball, its direction, height, the type of tactical application, and other factors. For example, a player who normally steps forward and turns to the side to play a stroke may, during a match or training, hit a forehand stroke across without executing all parts of the stroke (e.g., by not taking the racket back far enough, or by not executing the post-swing action) due to a change in the speed or direction of the incoming ball. Therefore, instantaneous observations and evaluations of hits may not always yield reliable results. Considering the influence of personal style and practice in the application of technique, there will likely be limitations and difficulties in scientific studies in this area. In this study, we provided appropriate conditions to observe the pure technique of tennis players in an isolated environment, in which they were unaffected by the opponent and the relevant decision-making mechanisms. Observational processes, which are an important information-gathering tool in sport in general, retain their importance despite multiple and rapid developments in technology.

Observational methods are among the most commonly used by researchers for matching and notational analysis (20). The results of the GTATT showed that the observations of the three coaches, who had observed the players over two weeks and assessed their technique by using our tool, were significantly correlated.

We can thus conclude that the proposed GTATT is highly reliable and can be used by tennis coaches in the field. The observers who participated in our experiment were coaches with at least 10 years of professional experience in the TTF and had a level-3 training license (senior coach) for coaching 10–12-year-old children. Their professional experience undoubtedly contributed to the high and significant correlation among their observations. We also found that the values of the parameters of the male and female tennis players differed only in terms of the ICCO.

We think that the above difference was obtained because there are more players in the male division than in the female division. Šlosar et al. (12) developed a valid and reliable scale of assessment called the “Tennis Rating Score for Child Tennis Players” (TRSC) to evaluate the technique of players aged 6–12 years for three basic tennis strokes: the forehand, backhand, and service. They made video recordings of 60 players (30 frames per second) practicing these three strokes (21 forehand shots, 22 backhand shots, and 17 services). They were then evaluated on days 1 and 7 by five tennis coaches by using the TRSC. The tennis coaches developed an initial TRSC list consisting of 20 items. The final selection by 15 experts reduced the number of items to 15, to be scored on a five-point scale. While this is different from our 12-item tool developed here, we noted certain similarities between items in our list and that formulated by Šlosar et al. (preparation for the shot, acceleration of the racket, contact point, and finishing).

Myers et al. (21) used the Observational Tennis Serve Analysis (OTSA) tool in their research on 33 non-professional tennis players (18 men, 15 women and eight observer coaches). Developed by the OTSA Women's Tennis Association in collaboration with the Kentucky Shoulder Center (Lexington, KY). It is a field-based tool that can be used to assess the mechanics of the tennis serve and offers the possibility to visually assess the mechanics of the service without requiring expensive laboratory equipment. The OTSA was evaluated by expert coaches, who examined the mechanics of the serve by using a video recording system. A concordance analysis between the raters was conducted one day apart. The interval between evaluations was one day (seven days in our study). The average values of kappa of the eight observers were moderate and high for all nine components of the OTSA. Their kappa values were in moderate agreement or better for eight of the nine components.

Torres-Luque et al. (20) analyzed all shots played in three matches at the 2014 Tennis Masters Cup in a study, in which they designed and validated a tool for the observational measurement of technical–tactical actions in singles tennis. Similarly to our study, the measurement tool was developed in five stages. Different expert groups were used to design and validity studies of the tool and test its validity. A total of four observers and 23 experts participated in this process. The design of the tool consisted of five phases: (a) review of the scientific literature and definitions of the variables provided by experts, (b) pilot observation study, (c) qualitative and quantitative evaluations of the tool by experts, (d) review and validation of the tool by experts (content validity), and (e) observational training and reliability assessment. In the context of the validity of the content of the tool, the value of Aiken's *V* was 0.94 and the reliability score was 0.81 according to the minimum values of *V*. The value of *V* was used as a criterion to determine whether a given item on the scale was statistically significant. Its value varied from zero to one, where values close to one indicated high content validity. The results showed that their designed tool provided valid and objective information on the technical–tactical movements of the players and their performance in singles tennis. Although our GTATT tool can be used to observe and evaluate players' technique in an isolated environment, the tool designed by Torres-Luque et al. can be used to assess both their technical and tactical choices by considering the entire game and can be used to evaluate the differences between winners and losers in a more holistic manner.

Studies conducted in recent years have also shown that there is a strong relationship between players' rankings and their performance in terms of playing the groundstroke. Research has shown that the accuracy of the velocity of the groundstroke significantly influences the performance of junior tennis players (22). Vergauwen et al. (23) assessed quality of the forehand groundstrokes of players in rally patterns in professional, low-, and intermediate-level tennis play while considering ball velocity– precision of ball placement (VP) and velocity–precision–success (VPS) index was calculated to reveal interactive effects. The validity and sensitivity of the ForeGround procedure in the target population were determined by verifying whether test scores reflected minor differences in tennis experience. They found that more experienced players scored significantly higher than beginner-level players in terms of the rate of success, ball velocity, and precision of ball placement as well as on the velocity–precision VP and the VPS indices. Our GTATT does not consider the speed and accuracy with which the player hits the ball. It can be used in combination with tracking and analysis software, like swingvision (which is available on cellphones), and videos. The GTATT is a useful tool when video recording is not available because it allows coaches to evaluate the quality of players' groundstrokes. Moreover, the use of tracking software that can use videos to provide feedback to players can help coaches fill out the 12 items on the GTATT.

Our study has certain limitations. We were unable to perform experiments on players aged 12–14 years, for which range of ages technical development is also very important, because we were unable to find an adequate number of participants. Moreover, we did not assess the anxiety of the subjects in our experiments before, during, and after the test to determine whether this had an effect on their stroke technique. Video recordings were not used by the observers/evaluators. In addition, we did not consider such parameters as the ball speed and the accuracy of the stroke in the GTATT. Future research in the area should use the GTATT in conjunction with tracking and analysis software, such as swing vision. Moreover, experiments should be performed on elite tennis players, with trainers with higher credentials and more experience recruited as observers. Finally, our measurement tool should be expanded to cover other strokes and forms of technique in tennis.

## 5 Conclusions

The GTATT developed in this study can be used by tennis coaches as a valid and reliable tool to assess the groundstroke technique of 10–12-year-old tennis players. The results of our experiments showed that improving the precision of their groundstrokes can help young tennis players improve their ranking and achieve competitive success, where this is consistent with the findings of previous studies. In addition, in this study, it was determined that there was a relationship between the technical abilities of young tennis players and their sensitivity, classification and competition success. This information can help tennis coaches plan effective training sessions for young players.

## Data Availability

The raw data supporting the conclusions of this article will be made available by the authors, without undue reservation.
